# Embedded trials within national clinical audit programmes: A qualitative interview study of enablers and barriers

**DOI:** 10.1177/13558196211044321

**Published:** 2021-12-09

**Authors:** Sarah Alderson, Thomas A Willis, Su Wood, Fabiana Lorencatto, Jill Francis, Noah Ivers, Jeremy Grimshaw, Robbie Foy

**Affiliations:** 1 Associate Professor in Primary Care, 120727 Leeds Institute of Health Sciences, University of Leeds, UK; 2 Senior Research Fellow in Primary Care, 120727 Leeds Institute of Health Sciences, University of Leeds, UK; 3 Research Fellow in Primary Care, 120727 Leeds Institute of Health Sciences, University of Leeds, UK; 4 Research Lead, 4919 Centre for Behaviour Change, University College London, UK; 5 Emeritus Professor, School of Health Sciences, City University of London, UK; 6 Professor of Implementation Science, 448446 School of Health Sciences, University of Melbourne, Australia; 7 Scientist, Department of Family Medicine, Women’s College Research Institute and Institute for Health System Solutions and Virtual Care, 7985 Women’s College Hospital, Toronto, Canada; 8 Associate Professor, Department of Family and Community Medicine and Institute of Health Policy, Management and Evaluation, University of Toronto, Canada; 9 Senior Scientist, Clinical Epidemiology, Ottawa Hospital Research Institute, Canada; 10 Professor, School of Epidemiology and Public Health, University of Ottawa, Canada; 11 Professor, Department of Medicine, University of Ottawa, Canada; 12 Clinical Professor of Primary Care, 4468 Leeds Institute of Health Sciences, University of Leeds, UK

**Keywords:** Quality improvement, clinical audit, embedded research

## Abstract

**Background:**

Audit and feedback entails systematic documentation of clinical performance based on explicit criteria or standards which is then fed back to professionals in a structured manner. There are potential significant returns on investment from partnerships between existing clinical audit programmes in coordinated programmes of research to test ways of improving the effect of their feedback to drive greater improvements in health care delivery and population outcomes. We explored barriers to and enablers of embedding audit and feedback trials within clinical audit programmes.

**Methods:**

We purposively recruited participants with varied experience in embedded trials in audit programmes. We conducted qualitative semi-structured interviews, guided by behavioural theory, with researchers, clinical audit programme staff and health care professionals. Recorded interviews were transcribed, and data coded and thematically analysed.

**Results:**

We interviewed 31 participants (9 feedback researchers, 14 audit staff and 8 healthcare professionals, many having dual roles). We identified barriers and enablers for all 14 theoretical domains but no relationship between domains and participant role. We identified four optimal conditions for sustainable collaboration from the perspectives of stakeholders: resources, that is, recognition that audit programmes need to create capacity to participate in research, and research must be adapted to fit within each programme’s constraints; logistics, namely, that partnerships need to address data sharing and audit quality, while securing research funding to ensure operational success; leadership, that is, enthusiastic and engaged audit programme leaders must motivate their team and engage local stakeholders; and relationships, meaning that trust between researchers and audit programmes must be established over time by identifying shared priorities and meeting each partner’s needs.

**Conclusion:**

Successfully embedding research within clinical audit programmes is likely to require compromise, logistical expertise, leadership and trusting relationships to overcome perceived risks and fully realise benefits.

## Background

There is growing interest in embedding trials within quality improvement programmes to enhance their impact while also generating robust evidence on what works.^1–3^ Large-scale audit and feedback programmes, which aim to improve patient care by reviewing clinical performance against explicit standards and directing action towards areas not meeting those standards, offer a prime opportunity for such experimentation. Audit and feedback was found to have modest effects on patient processes of care, with wide variation in effect sizes, from large, positive effects on quality of care to negative or null effects.^4^ There is relative paucity of head-to-head comparisons of different methods of providing feedback (e.g. varying comparators or feedback displays),^4^ with lack of robust empirical evidence for selecting one feedback method over another one.

Audit and other quality improvement programmes are often under pressure ‘to do something’, often with limited access to theoretical and empirical content expertise on how to optimise impact. National audit programmes may make incremental changes over time to how they deliver feedback and then observe any improvements in adherence to audit standards. But it is difficult to judge the impact of such changes given that any improvement effect is likely to be small and other factors may influence adherence, such as wider health service organisational reforms or (as an extreme example) a major pandemic. Using a rigorous evaluation design, specifically randomised controlled trials with parallel process evaluations, increases confidence in causal attribution. Embedding trials of different methods of delivering feedback offer a strategy to test approaches and to drive greater improvements in health care delivery and population outcomes. The large scale of national audit programmes makes it more likely that small to modest effects can be detected in a trial, with audit data themselves serving as trial outcomes (i.e. did practice change?) offering research efficiency.

We have previously proposed ‘implementation laboratories’ which embed research within existing large-scale initiatives such as clinical audit programmes.^5^ Implementation laboratories involve collaborations between health care organisations providing audit and feedback at scale, and researchers embedding head-to-head trials into routine quality improvement programmes. One example is the AFFINITIE partnership with the National Comparative Audit of Blood Transfusion in England.^6^ It randomised hospitals to two empirically- and theoretically-informed feedback interventions, which enhanced either the content of feedback reports or support to help hospitals act on feedback, and used audit data to assess effects on evidence-based blood transfusion practice. One other example is the Ontario Healthcare Implementation Laboratory, which aims to improve the impact of performance reports in nursing homes through randomising prescribers to different comparators (e.g. overall provincial average versus the top 25th percentile) and different ways of framing content (e.g. informing recipients that they have prescribed potentially harmful medications to 15% of their patients vs. avoided prescription-related harms in 85% of their patients).^7^ Establishing such implementation laboratories requires work in, for example, negotiating shared understandings, expectations and ground rules.^8^ They can also learn from other research-practice partnerships^9,10^ and develop infrastructure and working methods to sustain collaborations.^8^ However, there is limited experience of how to develop and run implementation laboratories, and none yet which have embedded trials evaluating different interventions in sequence.^5,6^

Moving towards a model of embedded trials will require changes in professional and organisational behaviours. It requires an understanding of the likely challenges and levers for change from the perspectives of different stakeholders. This study sought to generate evidence to inform this understanding. Specifically, we explored the perceived opportunities, costs and benefits of large-scale audit programme participation in long-term collaborations to improve audits through a programme of trials. We drew on the Theoretical Domains Framework^11^ as a behavioural framework for exploring different individual, socio-cultural and environmental barriers and enablers to change, with a focus on factors that are potentially amenable to change.

## Methods

This was an international, qualitative interview study eliciting perspectives and experiences on embedded trials within large-scale clinical audits.

### Study participants

We undertook purposive and snowball sampling to achieve a heterogeneous sample of (i) researchers with current or recent (within past three years) experience of conducting audit and feedback research; (ii) clinical audit programme staff who, at the time of the study, led or commissioned clinical audits as well as those potentially involved in feedback modifications, such as data managers and statisticians; and (iii) recipients of feedback (usually health care professionals). We aimed for 10 participants from each of these three groups,^12^ with varying experience of participation in research evaluating feedback interventions. We deliberately included participants with little or no experience of embedded trials because their expectations and concerns are important to understand when planning new programmes. Participants were identified through existing networks,^13^ beginning with delegates at an audit and feedback international symposium,^14^ clinical audit leads involved in known programmes of research and the Health Quality Improvement Partnership, which commissions the UK National Clinical Audit and Patient Outcomes Programme (NCAPOP).^15^ After identifying gaps in recruitment (e.g. health care professionals targeted by feedback), we initiated snowball sampling and asked interview participants for suggestions. The final number of study participants was guided by evidence of thematic data saturation, using a stopping rule of retrospectively checking that no new themes were identified in the final three interviews.^12^

### Development of interview schedule

SA, TAW, RF and FL developed a semi-structured interview topic guide (Online Supplement 1) that drew on our previous work and earlier experiences of research practice partnerships.^6,16–18^ Questions were structured around the domains of the Theoretical Domains Framework, representing a range of individual (e.g. knowledge, beliefs about consequences), socio-cultural (i.e. social influences, role and identity), and environmental (e.g. context and resources) barriers and enablers to behaviour change.^19^ This ensured a comprehensive exploration of behaviours involved in planning and conducting audit and feedback research which could potentially be amenable to change.^19^ We discussed and refined the topic guide with a reference group comprising individuals from national audit, clinical, behavioural science and research backgrounds, together with a panel of patient representatives that we routinely consult to ensure that our work addressed the public interest. We made no changes to the interview guide after piloting and analysing three interviews.

### Data collection and analysis

SA, TAW and SW conducted interviews during May to October 2019. They were completed face-to-face, by video or by telephone, according to participant preference. All interviews were audio-recorded and transcribed verbatim by a secretary. Interviewers checked each transcript against the original audio recording to ensure accuracy and to familiarise themselves with the data. Transcriptions were imported into NVivo 12 (QSR International Pty Ltd, Version 12, 2018) and anonymised. We used thematic analysis to identify experiences shared by participants.^20^ We analysed data by coding transcripts into the Theoretical Domains Framework in a recursive process.^20^ SA, TAW and SW independently coded data from interviews they had conducted and assigned initial codes before assigning each code to a theoretical domain. All codes within each domain were reviewed and a coding framework agreed. Differences were resolved through discussion with RF and FL. We also conducted further inductive analysis to check for any other beliefs not accounted for by the framework. We generated overarching themes by combining and comparing codes, then mapping how codes related to each other. It was noted whether subthemes arose solely among researchers, audit staff or health care recipients, or shared by all three. We reviewed the coherence of each theme to check the fit within all included codes. We finally defined each theme and its contribution to encapsulate participants’ experiences of barriers and enablers to embedding audit and feedback research within national clinical audits (see Online Supplement for an illustrative example).

Rigour of data analysis was ensured by several means. The research team comprised multidisciplinary members, including clinical academics with implementation science expertise and targeted by feedback programmes as clinical academics (SA, SW, RF), a behavioural scientist (FL), and an implementation scientist (TAW), all with experience of designing and conducting research with national clinical audit programmes and of applying the Theoretical Domains Framework. This allowed for investigator and theoretical triangulation of data analysis and interpretation. Where the researchers were familiar with examples of embedded research in large-scale clinical audits, interpretation was discussed with the other analysts to ensure codes were developed from the data and not the researchers’ own experience. We referred to Standards for Reporting Qualitative Research (SRQR)^21^ and reflected on these to ensure methodological rigour and trustworthiness.

## Results

We interviewed 31 participants, including 9 feedback researchers, 14 staff working on clinical audit programmes and 8 health care professionals, although many participants had dual roles and were analysed as both (for example, clinicians who also worked in national clinical audits) (Table 1). Compared with the proposed stopping rule of no new themes in the final three interviews, we reached saturation at 27 out of 31 interviews (Online Supplement S1).

**Table 1.** Study participants.

Participant characteristics	Number (%)
Country Affiliation or Base	
United Kingdom	26 (83.9)
Netherlands	1 (3.2)
Canada	2 (6.4)
United States of America	1 (3.2)
Australia	1 (3.2)
Role	
Feedback researcher	8 (25.8)
Feedback researcher and audit staff	1 (3.2)
Feedback researcher and health care professional	1 (3.2)
Audit staff	13 (41.9)
Audit staff and health care professional	4 (12.9)
Health care professional	4 (12.9)
Experience of embedded experimentation	
Yes	17 (54.8)
No	14 (45.2)

Tables 2 and 3 show the relationship between theoretical domains and themes. We found perceived influences on embedding experimentation in large-scale audit programmes in all 14 theoretical domains.

**Table 2.** Determinants of behaviour for domains within the Theoretical Domains Framework.

Theoretical domain	1. Knowledge	2. Skills	3. Social and professional role and identity	4. Beliefs about capabilities	5. Optimism	6. Beliefs about consequences	7. Reinforcement
Themes contributed to	Resources Logistical expertise Leadership Opportunities and benefits	Resources Logistical expertise Leadership Relationships Opportunities and benefits	Resources Logistical expertise Leadership Relationships Perceived risks Opportunities and benefits	Resources Logistical expertise Leadership Relationships Opportunities and benefits	Leadership Perceived risks Opportunities and benefits	Logistics Leadership Relationships Perceived risks Opportunities and benefits	Logistics Leadership Relationships Perceived risks Opportunities and benefits
Barrier/enabler to embedded research	Enablers more than Barriers	Enablers more than Barriers	Mixed	Mixed	Mixed	Mixed	Enablers more than Barriers
Number of transcripts coded to domain	27	22	24	27	19	31	27
*Illustrative quotes*	“I kinda realised there was a real evidence gap of like what actually is best practice audit reporting; and the needs of different people are very different…” *P23 – Healthcare professional and audit staff* “It was a quite naïve approach from the start 25 years ago, just sending reports and thinking that something magical would happen. And since maybe 5 to 10 years we started to more thoroughly think about what can make it really happen, that people will start improvement activities.” *P9 – Feedback researcher*	“I like the idea of […] advocating for providers and […] leveraging the fact that they, their information needs have to be met for, a feedback report to be useful.” *P10 – Feedback researcher* “I would say the programs strengths […] are: they’ve mostly got good clinical leadership. They’ve got excellent understanding of their data; and they’ve got excellent statistical folk. They’ve often got good IT folk who can build a platform but they’ll build what they’re asked to build […] they have a good instinctive grasp of how people use data in practice. But […] I think the nuanced side of things can be challenging.” *P12 – Audit staff*	*“I can imagine myself being really committed to [embedding research] and saying actually the benefits do outweigh it. I’m prepared to put the effort in at the start. Or to get all the niggly annoying things done because I think that this, the fact that it would be more efficient, more nimble, improved outcomes is worth it. But I think you could just as equally find someone who says no.”* *P9 – Audit staff* “I like doing this type of embedded research for a lot of different reasons, it’s just what I’m passionate about is doing something with the NHS and doing something removed from service just would not be interesting to me at all.[…] I find that motivating personally and I can definitely see the benefits.” *P20 – Feedback researcher*	*“I think the national clinic audits are an under exploited resource…* *I don’t think they’ve ever quite got the credit they should’ve had for the improvements that they’ve been doing; but it seems to me they provide an architecture for doing studies that have experimental designs.”* *P17 – Feedback researcher* “I don’t think there’s anything lacking here to my knowledge, […] in or that they don’t have access to partnering with expertise if they needed to. So I’m imagining within the research field of audit feedback there are clearly experts in that niche; […] they may not be like permanently based at [audit programme] but they would need to be for that necessarily.” *P1 – Audit staff and healthcare professional*	*“So I think the benefits are that we’ll stop commissioning things that we think are great, and start commissioning more of what we know is working.”* *P12 – Audit staff* “I think to be honest based on that experience, that is my main concern that you know we, we’ve been trying to optimise all the feedback and I think we now know how to do that more or less but […] if people simply don’t try sincerely to do anything then it’ll stop there but look, it will always look like all the feedback is not effective … That translation into action that’s, that could be a little challenge I find.” *P18 – Feedback researcher*	*“They were very open about being worried about what we would find. They have pressures of their own around the commissioning of the audit and the reputation of their organization […] I think they were worried about was if we found that their audit wasn’t making a difference or if there was an early warning rejection of the audits.”* *P11 – Feedback researcher* “I look at the [audit programme] reports and there’s masses of data in there. So trying to present that in a slightly different way and trying to present it in a more intelligent way and perhaps filtering out a lot of the stuff that doesn’t necessarily need to be presented, I can see definitely be a benefit for practices.” *P16 – Healthcare professional*	*“I think that’s equally important about how we can improve patient care through our routinely collected data. […] and so I think that in terms of overall resource allocation you know that it can be very cost effective, […] compared to say doing a big randomized controlled trial of a certain drug or a certain ventilator or whatever it is. So if you are collecting routine data at scale across the service overall and then feeding that back, then that in itself has a potential to have great patient impact.”* *P1 – Healthcare professional and audit staff* “You could imagine if they establish highly effective collaborations with researchers and they’re beginning to show that you’re not only getting service improvement but you’re getting a contribution to scientific history that could help stabilise them a bit and have them make their own business case more effectively.” *P17 – Feedback researcher*

**Table 3.** Determinants of behaviour for domains within the Theoretical Domains Framework, continued.

Theoretical domain	8. Intetions	9. Goals	10. Memory, attention and decision making processes	11. Environmental context and resources	12. Social influences	13. Emotion	14. Behavioural regulation
Themes contributed to	Relationships Opportunities and benefits	Logistics Relationships Opportunities and benefits	Resources Logistical expertise Relationships Opportunities and benefits	Resources Logistics Relationships Perceived risks Opportunities and benefits	Logistics Leadership Relationships Perceived risks Opportunities and benefits	Resources Logistics Leadership Relationships Perceived risks	Relationships
Barrier/enabler to embedded research	Mixed	Mixed	Barriers more than Enablers	Barriers more than Enablers	Mixed	Barriers more than Enablers	Barrier
Total number or transcripts coded to domain	5	19	11	31	28	11	1
*Quotes*	“The way we choose a clinical audit lead you’d expect would be somebody with expertise, somebody with interest, somebody with time. No. Where we choose a clinical lead is: ‘Who’s turn is it next?’ and it doesn’t matter what you know, what you do, what you can do. That’s not important. ‘Have you had a go yet?’ ‘No.’ ‘It’s your turn.’ […]The focuses is on the eye of the conference in Toronto. It’s not, ‘How can I make practice, practice better’.” *P6 – Audit staff* “They think that they’ve found […] the learning valuable and I think it will carry on.” *P3 –Feedback researcher and audit staff*	*“So I would love to do more! Absolutely love to do more. […] and we have […]some tentative discussions […] with [feedback researcher] and colleagues about trying to do something* *P15 – Audit staff* “So even an overall programme at [Audit programme], if we look back and see a change of six or seven percent that’s a pretty wildly successful programme, and so the idea that you can fairly simply once you’ve got it set up, […] tweak your […] design to get it more and more and more effective, even if it’s in the order of you know half a percent or a percent is actually to a programme like [Audit programme], not only is it successful in terms of it equals tens of millions of dollars over time, of impact. But also you know it does eventually equal health outcomes, you know if you do it right.” *P5 – Audit staff and feedback researcher*	“We are spending across the program a lot of time at the moment developing new visualisations, without to my knowledge a very strong evidence-base in the real world. I’m sure there’s lots of theoretical stuff out there So for example, from my perspective, in my work, in my role, I can’t commission any sort of technical spec for data visualisation at the moment because I don’t have the evidence that says what a good example is this thing.” *P12 – Audit staff* “Officially I am not resourced to do the research part. So it is kind of finding a way to make some fundings or resources […] available for doing the research.” *P9 – Feedback researcher*	*“So I would put data sharing agreements and data quality near the top of the list of challenge. To do a multi-centre project where you are collecting data from many centres and sharing it and using that data for audit and feedback, every single centre requires a data sharing agreement with a common repository of data and secondly the quality of the data may vary between centres which makes it very difficult for inter-centre comparisons […] so that’s a major challenge*” *P30 – Feedback researcher and healthcare professional* “So I would be strongly in support of the idea of doing it without […] signed up consent. Or ethically necessary. I mean you’re right. It probably would be a trial killer as well. Cos […] it would be very difficult to manage that.” *P14 – Audit staff and healthcare professional*	*“We need to have a continued brand awareness and we need to keep people happy to a degree. So a GP needs to go “oh yeah, I like that group, they do stuff that really resonates with me and they do stuff that I find acceptable and so therefore I’ll continue to engage with them’. So for example, when I started talking about playing with the valence of the messages people were saying “what do you mean you’re gonna have a negative message? If people open up a report and they see a negative message they’ll never open up one of our reports again.”* *P5 – Audit staff* *“*S*ometimes it’s really hard to find that person though or that person is just like really busy or they change and somebody else comes in and they don’t know the thing so well and […] you know that’s the same whether it is national clinical audits or anything else.”* *P25 – Feedback researcher*	“We have feedback theories that tell us […] receiving feedback is emotional. […] there are harms however mild […] there are unintended consequences let’s say of feedback. And so I think it’s a new area where we have sorted through […] what the harms and benefits are to an adequate level […] and so maybe there’s some, just my own anxiety around you know what are we doing?” *P10 – Feedback researcher* “I mean actually it’s fun. We have a really good time; it’s exciting, it’s an environment, I think, that people can express ideas […] that are seriously considered and solutions are found to problems really.” *P7 – Audit staff*	“I think … partly because I’m interested in the tailoring and adapting feedback […] the challenge I noticed myself, focused on is this trade-off between customising, personalising and adapting […] everything versus developing something that’s efficient, standardised and useful […] to other people. So that would be one challenge…” *P10 – Feedback researcher*

Thematic analysis indicated that there was no association between theoretical domains and participants’ roles. In the following, we report on six overarching themes which we elaborated and mapped onto theoretical domains (presented in italics): resources, logistics, leadership, relationships, perceived risks and opportunities and benefits. Key barriers to embedding experimentation fell within three main domains: memory, attention and decision-making processes; environmental context and resources; and emotion. Key enablers fell mostly within three domains: knowledge, skills and reinforcement. There was a high level of agreement across roles, including those with dual roles, for all themes.

### Resources

Clinical audit staff generally noted that their small, already resource-poor teams limited their ability to take part in audit and feedback research *(Environmental context and resources)*, and that existing competing priorities were too overwhelming to consider further commitments *(Memory, attention and decision-making processes)*. They described various funding models of different clinical audit programmes and that many were run or staffed by volunteers *(Beliefs about capabilities)*. Many audit staff said they felt making changes to the audit or feedback reports in practice were resource-intensive and there was a risk of overstretching teams in taking on more work *(Environmental context and resources; Emotion)*. The majority of audit staff we interviewed said that insufficient funding of the audit was a considerable constraint for taking part in a research collaborative (*Environmental context and resources*).

There’s a lot of audits that are running on a shoestring as well! So a lot of people that want to improve what they do, you know I’m talking about in terms of delivering their audit … but they’re running on a shoestring financially. *(P15, Audit staff)*

A minority of audit staff described how their current audit programmes worked with clinicians and external parties for short periods only, such as one audit cycle, making continuity for research difficult *(Environmental context and resources)*. They described how clinicians’ roles in identifying audit criteria were sometimes seen as an opportunity to further their own research and leadership profiles, rather than improve health care or effectiveness of the audit programme *(Social and professional role and identity)*. Audit staff further noted how they often worked to strict timelines for delivering feedback, whereas researchers were restricted by funding cycles. They all recognised that collaborative research needed to understand and fit within these constraints *(Environmental context and resources)*.

You are having to align fairly complex research governance processes with those external deadlines and that … is definitely a challenge! *(P11, Feedback researcher and audit staff)*

Audit staff working in programmes with smaller budgets and less funding noted that researchers had to recognise audit programme needs *(Skills)* and leverage research funds to enable evaluation *(Environmental context and resources)*. Researchers aiming to embed trials within national audit programmes had to understand such pressures. Collaborative partnerships were unlikely to be viable without additional, sustained funding and a willingness to align the design and conduct of trials to existing organisational practices.

### Logistics

Audit staff, researchers and health care professionals all mentioned multiple logistical challenges around embedding research within audit programmes, namely, data quality and sharing, commissioning cycles, coordinating multiple parties and lack of evidence-based performance measures. Ethical barriers were not mentioned and one researcher commented (P17 – feedback researcher) that not embedding trials might actually be considered unethical as feedback methods were typically changed without formal evaluation *(Environmental context and resources)*. Data sharing was not seen to be a challenge for most UK-based audit programmes with audit staff reporting data sharing agreements in place that allowed research use of data without the need for additional research permissions, although international participants expressed less certainty regarding data ownership *(Environmental context and resources)*. Some audit staff described how their audit programme made their data and findings publicly available, making data sharing easier.

All feedback researchers described how longer-term, programmatic funding was difficult to obtain in comparison to shorter research projects, but needed to develop and test different ways of delivering feedback over two or more audit cycles *(Knowledge)*. However, those with experience of applying for funding found that embedded experimentation was not considered a ‘sexy’ topic or a priority for funders. Convincing funding panels of the need for such research was seen as a major barrier *(Knowledge)*, despite potential benefits for population health care *(Goals; Environmental context and resources)*. Both researchers and audit staff recognised that where clinical trial units had been previously involved, this contributed to high research costs.

I don’t think any funders would consider it may be sexy for instance? And might, well if not be aware on panels, I don’t think they often will be aware of […] the area. *(P18, Feedback researcher)*

Most researchers with experience in embedding research described how embedded experimentation depended on the availability of both credible data and evidence-based audit standards. Reliable data collection and data quality for trials presented common challenges for audit staff and feedback researchers; some audit staff highlighted data limitations affecting other audit programmes *(Skills; Belief about capabilities)*. One audit staff participant (P6) explained how taking part in embedded research raised awareness of data quality problems in their own programme, empowering them to make changes to the audit *(Social and professional role and identity)*. Some audit staff described how their audit programmes struggled with a lack of evidence-based standards on which to assess health care providers *(Beliefs about capabilities)*. Their standards were set pragmatically, based more on current achievement than on rigorous evidence. They wanted research to prioritise establishing validated standards before planning embedded experimentation. It was argued that using contentious standards as outcome variables may risk resistance from health care professionals *(Goals)* and potential dismissal of the audit.

No one knows what the, what a good induction rate is or a good elective caesarean section rate is so, that’s quite tricky to then work with. Some would say well the audit could pick one. But then I would anticipate we would have a lot of backlash from people. Some people would say you’re too high; some people, you’re too low and everything in-between. *(P21, Audit staff)*

A minority of researchers described further logistical issues regarding constraints of audit data derived from electronic health records, such as providers using third party record systems that they are unable to change to collect data needed for the audit *(Environmental context and resources)*. There were also concerns that participants would share feedback with one another, risking contamination between trial arms *(Social influences)*. Continued organisational restructuring had the potential to disrupt cluster randomised trials if units of randomisation were subject to mergers, such as ongoing mergers of general practices into larger practices in UK primary care *(Environmental context and resources)*. Successful embedded research required stable relationships and organisations. Participants with and without previous experience of embedding research within clinical audits identified such logistical challenges. There was a recognition that all parties should review and agree processes for data extraction, sharing, checking and cleaning before commencing embedded research.

### Leadership

Feedback researchers and audit staff highlighted both the leadership skills and enthusiasm for research of the audit programme leader as critical success factors in a collaboration *(Skills; Social and professional role and identity)*. Researchers said they believed clinical audit leaders needed an understanding of equipoise around the most appropriate design, as well as of feedback methods *(Knowledge)*. We found that some audit staff and health care providers without experience of embedded research struggled with equipoise, expressing concerns that experimentation which involved removing aspects of feedback assumed as beneficial would degrade their feedback *(Beliefs about consequences)*.

All participants described that optimistic leadership motivated team involvement and that leaders’ personal interests in research encouraged a team culture of learning and inquisitiveness *(Skills; Optimism; Social and professional role and identity)*. Leaders had to be able to convince others, including audit programme and research funders and health care providers, of the need for research *(Optimism; Social and professional role and identity)*. Health care professional participants reported feeling encouraged to take part in feedback research with an enthusiastic and respected audit programme leader *(Skills; Belief about capabilities; Social influences)*.

They genuinely seem to have been interested and keen to learn from the findings. *(P11, Feedback researcher and audit staff)*

Most audit staff described that among local health care provider teams, key individuals’ understanding of what embedded feedback experimentation aims to achieve often depended on their enthusiasm for and commitment to audit programmes *(Knowledge; Social influences; Reinforcement)*. All participants described how a steer from local provider and purchasers of health care leadership could strengthen involvement and legitimise the research *(Skills; Social and professional role and identity)*.

So, you might have a clinical lead for [audit programme] locally in a [health care system] who is loving a current data visualisation and, you know that person changes at exactly the time, you know you switch the visualisation; and the fact, the drop off isn’t that the visualisation has gone, the drop-off is that you’ve lost the key person locally. *(P12, Audit staff)*

Audit staff participants’ typically minimal experience of embedded research was important in this theme, with misunderstanding of research equipoise in feedback research and the role of the control group in trials. Identifying an enthusiastic leader to engage audit team and health care providers was considered helpful by those with and without experience of embedding research. Researchers needed to promote an understanding of research equipoise to ensure that negative trial results are not misrepresented as research failures or lack of audit impact to encourage successful partnerships.

### Relationships

Most audit staff and feedback researchers with experience of embedded research described how relationships and trust between audit programmes and researchers took time to develop *(Reinforcement; Social influences)*. Audit staff with and without experience described how they wanted to start slowly with simple studies to establish benefits and procedures so that they could balance their involvement with other competing demands *(Environmental context and resources, Reinforcement)*.

Diplomatic skills *(Skills; Intention)* were described as essential to maintain the relationship when difficulties arose, particularly by audit staff and researchers with prior experience to embedding research in clinical audits.

So the kind of skills I need are a bit of diplomacy, a bit of prompting, a bit of time management, a bit of sort of people management in that respect, but also the ability to step back and not say ‘this is what I think we should do’. […] and then just tenacity […] in diplomacy again to smooth over some troubled waters, bits and pieces, keep going. *(P6, Audit staff)*

There was apprehension over losing control by those with previous experience in embedded research, but also identified by those considering participation: researchers wanted more control over data gathering and audit staff wanted to retain control over audit content *(Beliefs about consequences; Memory, attention and decision making processes)*. All participants described how being involved needed patience, particularly in setting up research *(Skills; Social influences)*. Co-design and involving health care participants in the research was mentioned by some audit staff and health care professionals as a means to build trust between the researchers, audit programmes and health care system *(Goals; Social influences)*.

The clinical teams, you know, may be interested in improvement but often they’re interested in doing their clinical work and not being bothered too much. And the clinical audit leadership wants to demonstrate that the audit is, is worth it and that it’s producing value. So I can see that that would be one of the first tasks is umm, is reaching a shared understanding. *(P17, Feedback researcher)*

All participants agreed that shared priorities in improving the effectiveness of clinical audit programmes meant that benefits of embedding experimentation in existing programmes outweighed the challenges for all *(Intentions; Goals)*. Audit staff and health care professionals noted that involvement should not be too onerous and there had to be a balance between research rigour and pragmatic decisions *(Beliefs about capabilities)*. Where this had previously occurred, researchers and audit staff valued a sustained relationship *(Reinforcement)*.

Early identification and agreement of shared priorities for both the research and the clinical audit programme would allay some apprehension over losing control, as would starting with small changes to the feedback to avoid alienating end users before considering tackling more complex or larger changes.

### Perceived risks

The majority of audit staff and health care professionals raised concerns about negative unintended consequences *(Beliefs about consequences; Emotion)* to taking part in embedded research. They were concerned that the funding and renewing of audit programmes could be threatened where they fail to demonstrate improved effectiveness in embedded trials *(Beliefs about consequences; Environmental context and resources),* with subsequent loss of employment. Some audit staff noted that it would be difficult to demonstrate improved impacts of experimental feedback methods because of ‘ceiling effects’ associated with pre-existing high levels of performance, where only marginal improvements could be made in care.

They were very open about being worried about what we would find. They have pressures of their own around the commissioning of the audit and the reputation of their organisation. *(P11, Feedback researcher and audit staff)*

Some audit staff and feedback researchers were concerned about damaging the relationship with health care providers *(Beliefs about consequences)* by changing the format or design of feedback. As described earlier, this could alienate and disengage end users and so undermine the audit programme and the research *(Reinforcement; Beliefs about consequences)*. Audit staff in particular felt that protecting the audit programme brand was important *(Social influences)*.

People get used to our reporting format. They get, they finally got, got that now! You know I understand what that’s showing me now! We go “Wee!” We’ve changed it! You know like, no, so what we might think is terribly good in their space, they might go “God I don’t understand it now!” You know back to square one! *(P15, Audit staff)*

Most participants across all roles described this type of research as low-risk and low-cost to health care providers *(Beliefs about consequences; Reinforcement)* although audit staff noted that balancing the needs of all stakeholders and third-party involvement was a significant challenge *(Social influences)*.

The ‘branding’ of the clinical audit programmes and its existing relationship with their recipients was strongly promoted by all audit staff interviewed. Changes to the feedback, or the wrong choice in audit standards could have a large impact on the future of the clinical audit programme. Audit standards had to be chosen carefully for feedback research to ensure they were underpinned by a strong evidence base and that there was scope for improvement. This required researchers to balance research ambitions with pragmatic decisions to enable research participation by clinical audit programmes.

### Opportunities and benefits

All feedback researchers, audit staff and health care professionals gave examples of how clinical audit programmes might benefit from embedding feedback trials in research collaborations *(Optimism; Beliefs about consequences)*. These included opportunities to gain new skills and new ideas about how to improve audit programmes, increased funding, and further opportunities from new collaborations *(Skills; Beliefs about consequences; Reinforcement)*.

When we retender for running the national clinical audits, it’s useful to have an evidence base on where we’re going for focus. We want to do lots of things, we’re limited in terms of capacity in what we can realistically implement. So knowing that we’re implementing something that’s going to make more of a difference and then have a knock on impact hopefully on patients. *(P4, Audit staff)*

Audit staff considered increasing their audit programme’s effectiveness as integral to their roles: embedding this type of research was a strategic decision that allowed them to raise awareness of the clinical audit and its team, satisfy funders that improvement work was ongoing, demonstrate the programme’s impact, and help improve patient care *(Social and professional role and identity; Goals)*. All participants, including those with and without experience, considered that embedded experimentation could bring about as much benefit for health care systems and patients as clinical research. Audit programmes were an under-used research resource *(Goals; Intentions; Beliefs about capabilities)* and embedding research within an existing structure represented an efficient model of quality improvement and improved the evidence-base for audit and feedback *(Reinforcement; Memory, attention and decision-making processes)*.

It’s likely that the new discoveries are likely to plateau and really now the bigger challenges putting into effect the medicines and treatments that we know work, I think has gotta be the kind of highest priority really because there’s not really any point in developing new treatments if we’re not using the ones we have currently as effectively as we could. *(P23, Audit staff and health care professional)*

Most audit staff and health care professionals were generally keen to be involved *(Goals; Emotion; Optimism)*. Participants perceived benefits of strengthening the evidence for recommissioning of audit programmes, securing funding for future research and, most importantly, the potential for significant population health benefits (*Beliefs about consequences*).

Box 1 summarises what we describe as ten ‘top tips’ that we identified from our findings for the creation of successful collaborations between audit programmes and feedback researchers. Thus, audit programmes need capacity to take part in research, with adequate resources and staffing to make changes to feedback *(top tip 1)*, which need to occur in the context of an audit’s and researcher’s time-limited constraints *(2)*. Further, logistical issues regarding data sharing and quality, research funding and trial contamination need to be resolved *(3)*. Enthusiastic and engaged audit programme leaders are needed who can motivate a research-interested team as well as engage local health care leaders *(4)* and understand research equipoise *(5)*. Collaborations between research teams and audit programme staff need to be underpinned by trusting and sustained relationships through identifying shared priorities *(6)* and balancing research and pragmatic imperatives *(7)*. To reduce perceived risks of participation in embedded experimentation, audit standards need to be evidence-based to ensure engagement of clinicians *(8)*, while also research desires should be balanced with pragmatic changes to feedback *(9)*. Finally, there is a need for all stakeholders to recognise the potential benefits of successful collaborations between audit programmes and feedback researchers, such as improving population health, increased investment, ongoing relationships and demonstration of impact to audit and research funders; however, this message needs to be recognised by all stakeholders *(10)*.

**Box 1.** Ten ‘top tips’ for the creation of successful collaborations between audit programmes and feedback researchers.

*Resources*
1. Consider what extra resources the audit programme(s) will need
2. Agree timelines with both research and audit team
*Logistics*
3. Review and agree processes for data extraction, sharing, checking and cleaning
*Leadership*
4. Identify an enthusiastic leader to engage audit team and healthcare providers
5. Promote an understanding of equipoise to ensure that negative trial results are not misrepresented as research failures or lack of audit impact
*Relationships*
6. Ensure and agree shared priorities for research and clinical audit programme
7. Start with small changes to avoid alienating end-users before tackling more complex or larger changes
*Perceived risks*
8. Choose audit standards carefully for feedback research, ensuring they are underpinned by a strong evidence base and that there is scope for improvement
9. Balance research ambitions with pragmatic actions
*Opportunities and benefits*
10. Recognise small improvements may have significant population benefits – message needs to be heard by funders, commissioners and health care system

## Discussion

This is the first in-depth exploration of issues around embedding research within large-scale audit programmes. Previous research has mainly focused on the use of audit data by clinicians to improve health care, or in clinical research (such as epidemiological studies).^22^ This qualitative study provides subjective evaluations of the impact of embedding research in audit programmes; it remains to be seen whether such embedded research really can deliver sustained, incremental improvements in health care. Our theory-guided approach allowed us to identify the cognitive, affective, social and environmental influences on the behaviour of key players involved in embedding research within audit programmes.

Overall, study participants believed that the benefits of participating in future collaborative research to improve feedback’s effectiveness outweighed the risks. These findings are particularly relevant for research funders, clinical commissioners, national audit leads, and health care quality improvement leads, as they have implications for future implementation laboratory design and evaluation.

### Strengths and limitations

Our purposive sampling strategy had limitations; the majority of health care staff recruited had some current or previous involvement with audit programmes and knew we had involvement in developing embedded trials, which is reflected in our interview guide, potentially subjecting our findings to social desirability bias. Despite this, study participants reflected in detail on the potential challenges of embedding research as well as the benefits. Our links with the Audit and Feedback ‘Metalab’^13^ international collaboration enabled us to leverage a reasonably diverse range of stakeholder perspectives and draw on examples of embedded research internationally. Participating health care professionals were mostly secondary care based. Recruitment of health care staff from primary care, where audit programmes are limited, ensured that we captured insights from those not involved with audit programmes. We included those without experience of embedding research to identify potential barriers that had not been resolved. All participant roles and those with and without experience of embedding research within audits identified similar optimal conditions, potential risks and benefits, suggesting that when developing major initiatives involving research-practice partnerships the majority of challenges are predictable and could be mitigated through communication and detailed planning.

The majority of our participants had experience with UK national clinical audit programmes, but our findings have implications for large-scale audit programmes and benchmarking health care data in other countries that aim to develop major initiatives involving research-practice partnerships to improve audits through embedding trials to evaluate specific feedback strategies.

### Comparison to existing literature

There is little evidence on embedding research in implementation laboratory settings. They are related to the ‘Learning Health System’ concept, which also involves integrating evaluation within routine care and rapid deployment through a continuous learning and improvement cycle.^23^ Very few descriptions of successful learning health systems have been published; however, initial experience suggests a need for adequate funding, robust data systems and an organisational culture that values quality improvement.^24^ We suggest that actively including audit programme partners in the research team can help overcome institutional pressures in the design phase for audit programmes already facing considerable financial and organisational challenges.

Our research augments work on stakeholder perspectives when aligning research and practice. Research-practice partnerships may provide structure and opportunity for developing a shared cognitive space around which collective action can be organised. Although time-consuming, a process of consensus building can deliver several benefits: aligned priorities; a trusting relationship though the relinquishing and sharing of power; and recognition of potential long‐term benefits of embedded trials within quality improvement programmes.^8^ Not embedding research into quality improvement initiatives within national clinical audits was seen as unethical by one participant. While this was a minority view, it is an important consideration reflected in other quality improvement literature.^25,26^ The difficulties of achieving, then sustaining, a partnership are similar in other contexts,^9^ with the need for appropriate structures (including leadership and establishing roles) and processes to facilitate optimal conditions for genuine and collaborative action.^8^

Embedding research in clinical audit programmes, in an implementation laboratory setting, has been suggested as a means of enhancing the impact of audit and feedback while also producing generalisable knowledge about how to optimise effectiveness.^5,22^ Embedding sequential head-to-head trials testing different feedback methods in an audit programme provides a robust empirical driver for change. Modifications identified as more effective than the current standard become the new standard; those that are ineffective are discarded. Testable recommendations for feedback modifications suggested by Brehaut et al.,^27^ such as using an average or high-performing comparator, have minimal cost implications; however, our study suggests there are resource implications for audit programmes that are not currently met. Marginal gains in audit and feedback effects, such as a one per cent gain in effectiveness, are likely to be worthwhile at a population level and achievable within an adequately resourced implementation laboratory.^5^ Funders of clinical audit programmes should consider added value from embedded trials to improve effectiveness whilst recognising its inherent logistical challenges.

## Conclusion

This study suggests that those leading and participating in audit programmes believe that the benefits of embedding feedback research outweigh the risks and challenges. There is willingness by audit staff and health care professionals in our study to participate in an implementation laboratory to enhance the impact of audit programmes while also producing generalisable knowledge about how to optimise audit and feedback effectiveness. We identified the optimal conditions for sustainable partnerships between clinical audit programmes and researchers in delivering collaborative research to improve the effects of feedback. Our findings can inform a set of ‘ground rules’ and recommendations on how to optimise conditions for sustainable collaboration between national audit programmes and researchers.

## Supplemental Material

sj-pdf-1-hsr-10.1177_13558196211044321 – Supplemental material for Embedded trials within national clinical audit programmes: A qualitative interview study of enablers and barriers Click here for additional data file. Supplemental material, sj-pdf-1-hsr-10.1177_13558196211044321 for Embedded trials within national clinical audit programmes: A qualitative interview study of enablers and barriers by Sarah Alderson, Tom A Willis, Su Wood, Fabiana Lorencatto, Jill Francis, Noah Ivers, Jeremy Grimshaw and Robbie Foy in Journal of Health Services Research & Policy
