# Supplementary material for: Embedded trials within national clinical audit programmes: A qualitative interview study of enablers and barriers
Source: J Health Serv Res Policy. 2021 Dec 9;27(1):50–61. doi: 10.1177/13558196211044321 (PMC8772016; doi:10.1177/13558196211044321)

Online Supplement 1

Illustrative interview topic guide: The opportunities, costs and benefits of embedding implementation research within national audit programmes

Before the interview starts:

- Brief overview and introduction to aim of study
- Checking of consent
- Checking for permission to record, including explanation that participant can stop at any time

About the participant:

- Job title and affiliation(s)
- Role(s)
- Experience & knowledge of audits, including national audits
- Experience & knowledge of research, including audit and feedback research

- ***What are your experiences or knowledge of conducting or taking part in audit and feedback research allied to an audit programme? (if relevant)***
    - Perceived need
    - Previous work
    - What it would involve
    - What drove them to take part
    - Interpersonal skills needed
    - Did they evaluate the success?
    - Dissemination
    - How easy / difficult (what was easy / difficult)

- ***What do you think the benefits are (or have been) of embedding audit and feedback research in national audits?***
    - Priorities? (Goals)
    - Priorities of colleagues
    - What are they trying to achieve (Reinforcers)

- ***What do you think the costs and resource implications are (or were) of embedding research in national audits?***
    - Time
    - Financial resources
    - Staffing
    - Knowledge / skill mix for success

- ***What are the challenges to embedding research (in national audits)?***
    - logistical issues,
    - alignment of timelines and human resources,
    - data sharing arrangements & data quality;

  - level and type of support needed to build sustainable enhancements
  - stability in healthcare partner systems
  - team composition & dynamics
  - clarifying roles & responsibilities
  - identifying shared priorities
  - optimising design
  - ethical considerations
  - funding
  - Competing priorities / importance in relation to other roles
  - Tension / pressures

- ***Do the benefits (pros) outweigh the challenges (cons)?***
  - Would they do it again?
  - Would they consider doing it?

- ***Is there anything else you feel it is important for us to know about embedding research in national audits?***

Thank participant for their time

Provide voucher and certificate, as appropriate

Figure S.1 – Data saturation of theoretical domains

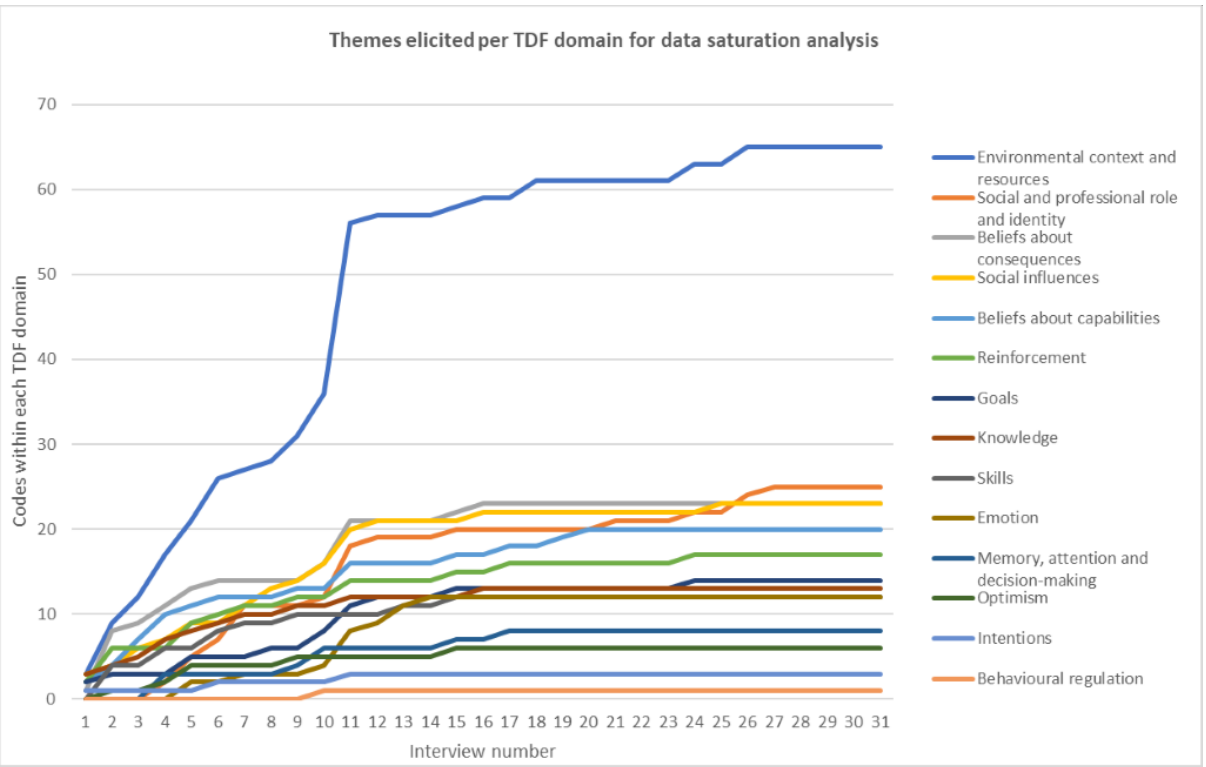

Supplement: sj-pdf-1-hsr-10.1177_13558196211044321 – Supplemental material for Embedded trials within national clinical audit programmes: A qualitative interview study of enablers and barriers [file sj-pdf-1-hsr-10.1177_13558196211044321.pdf]
